# Residual Antibiotics in Decontaminated Human Cardiovascular Tissues Intended for Transplantation and Risk of Falsely Negative Microbiological Analyses

**DOI:** 10.1371/journal.pone.0112679

**Published:** 2014-11-14

**Authors:** Marina Buzzi, Anna Guarino, Claudio Gatto, Sabrina Manara, Luca Dainese, Gianluca Polvani, Jana D'Amato Tóthová

**Affiliations:** 1 Cardiovascular Tissue Bank of Emilia-Romagna, Azienda Ospedaliero-Universitaria Policlinico S. Orsola-Malpighi, Bologna, Italy; 2 Cardiovascular Tissue Bank of Lombardia, Centro Cardiologico Monzino, Milan, Italy; 3 Research and Development department, AL.CHI.MI.A. S.r.l., Ponte San Nicolò, Italy; University of Pisa, Italy

## Abstract

We investigated the presence of antibiotics in cryopreserved cardiovascular tissues and cryopreservation media, after tissue decontamination with antibiotic cocktails, and the impact of antibiotic residues on standard tissue bank microbiological analyses. Sixteen cardiovascular tissues were decontaminated with bank-prepared cocktails and cryopreserved by two different tissue banks according to their standard operating procedures. Before and after decontamination, samples underwent microbiological analysis by standard tissue bank methods. Cryopreserved samples were tested again with and without the removal of antibiotic residues using a RESEP tube, after thawing. Presence of antibiotics in tissue homogenates and processing liquids was determined by a modified agar diffusion test. All cryopreserved tissue homogenates and cryopreservation media induced important inhibition zones on both *Staphylococcus aureus*- and *Pseudomonas aeruginosa*-seeded plates, immediately after thawing and at the end of the sterility test. The RESEP tube treatment markedly reduced or totally eliminated the antimicrobial activity of tested tissues and media. Based on standard tissue bank analysis, 50% of tissues were found positive for bacteria and/or fungi, before decontamination and 2 out of 16 tested samples (13%) still contained microorganisms after decontamination. After thawing, none of the 16 cryopreserved samples resulted positive with direct inoculum method. When the same samples were tested after removal of antibiotic residues, 8 out of 16 (50%) were contaminated. Antibiotic residues present in tissue allografts and processing liquids after decontamination may mask microbial contamination during microbiological analysis performed with standard tissue bank methods, thus resulting in false negatives.

## Introduction

Tissue banks, including cardiovascular tissue banks, procure, process, store, and distribute human donor tissues intended for transplantation. The main clinical applications of heart valve allografts include valve replacement in patients with congenital or acquired heart disease and substitution of infected heart valve prosthesis [1], [2]. Vascular allografts are used for vascular reconstruction, revascularization in absence of autogenic veins in patients with idiopathic or diabetic vasculopathies, and replacement of infected vascular prosthesis [1], [3].

Decontamination is a critical phase of the processing of human tissues intended for transplantation. Tissue banks worldwide have established a wide variety of homemade antibiotic cocktails and procedures to eliminate microbial contaminants from tissues to maximize the safety of allografts [2], [4]–[6]. Tissue decontamination may result in the presence of antibiotic residues in both decontaminated tissues and processing liquids [7].

Residual antibiotic concentrations may induce bacteriostasis of microorganisms eventually present on tissue samples and lead to false negative results during microbiological analysis [8]. According to the European and US Pharmacopoeias [9], [10], the sterility test must be preceded by complete removal of any possible interfering antimicrobial agents.

No specific standards for microbiological analysis in tissue banking are available. Consequently, each tissue bank shall validate the method for tissue and liquid sample microbiological analysis, which is essential to determine whether the tissue can be released for transplantation [11]–[13]. The microbiological analysis is usually performed by means of automated blood culture systems or direct inoculum of growth media [13], [14]. In general, these systems do not consider the presence of high antibiotic residues in tissues and processing liquids after decontamination, which is a possible cause of falsely negative microbiological results. This problem is further underestimated by the general assumption that antibiotics are rapidly degraded during the sustained tissue incubations necessary for sterility assessment.

To investigate the impact of antibiotic residues on microbiological analysis, we used human cardiovascular tissues, decontaminated with different antibiotic cocktails, two tissue bank microbiological methods and the RESEP tube method, which has been previously validated for the elimination of antibiotics from tissues and processing liquids, decontaminated with an industrial cocktail. All cryopreserved samples tested for sterility were also analyzed for the presence of antibiotic activity by a modified agar diffusion method.

## Materials and Methods

### Ethics statement

Donor tissues were collected and processed in compliance with the European Directives on setting quality standards and technical requirements for the processing of human tissues and cells. Only tissues unsuitable for transplantation were used for the study, and the permission was granted by the institutional review board of the Cardiovascular Tissue Bank of Emilia Romagna (CTBER) and the Cardiovascular Tissue Bank of Lombardia (CTBL). All clinical investigation has been conducted according to the principles expressed in the Declaration of Helsinki.

### Tissues procurement and processing

Sixteen human cardiovascular tissues (5 aortic heart valves and 11 blood vessels graded as unsuitable for transplantation due to anatomical defects) were included in the study. Six and 10 tissues were collected and processed by CTBER and CTBL, respectively.

Tissues were transported to the tissue banks at 4°C in antibiotic-free transport media, Celsior (Genzyme Europe BV, Netherlands; CTBER) or Eurocollins (S.A.L.F. S.p.A., Italy; CTBL), and processed following tissue bank standard operating procedures. In particular, tissue were decontaminated with tissue bank-prepared antibiotic cocktails, containing cefoxitin, lincomycin, colimycin, vancomycin in RPMI1640 (cocktail 1, CTBER) or cefotaxime, lincomycin, vancomycin, polymyxin B sulfate in RPMI1640 (cocktail 2, CTBL) at 4°C for 24 h (valves) and 72 h (vessels). Tissues were then rinsed twice for 1 min with NaCl 0.9% (only for CTBER) and finally cryopreserved in RPMI 1640 (Sigma-Aldrich, St. Louis, MO, USA) containing 10% DMSO (Wak-Chemie, Germany), using programmable freezers with freezing rate of −1°C/min. The CTBER cryopreservation solution also contained 2% human albumin (Kedrion Biopharmaceuticals, Italy). Cryopreserved samples were then transported in dry ice to the R&D department of AL.CHI.MI.A. S.r.l. for further microbiological analysis and detection of antibiotic residues.

### Microbiological analysis

Microbiological analysis of samples collected from processing liquids and tissues, before and after decontamination, was performed by the tissue banks, according to their standard operating procedures. At CTBER, 5 ml of the transport and cryopreservation solutions were introduced in the BacT/ALERT vials to detect aerobic and anaerobic bacteria and incubated at 37°C in the BacT/ALERT 3D automatic detection system (Biomérieux, France) for 5 days. Tissue samples were immersed in fluid thioglycollate medium (FTM) and tryptic soy broth (TSB) and incubated at 35°C for 7 days (direct inoculum method). At CTBL, 1 ml of transport and cryopreservation solutions and tissue samples were introduced in FTM and incubated at 37°C for 7 days (direct inoculum method). At R&D department of AL.CHI. MI.A S.r.l., 5 ml of thawed cryopreservation solutions and tissue samples were tested by incubation in TSB and FTM at 24°C and 33°C for 14 days, respectively (direct inoculum method, according to the European Pharmacopoeia [9]). In addition, separate samples from same solutions and tissues were introduced in the RESEP tube (AL.CHI.MI.A. S.r.l., Italy) containing a specific resin mixture, which was expressly formulated to eliminate the antibiotics contained in the BASE.128 decontamination medium (AL.CHI.MI.A. S.r.l., Italy). The RESEP tubes were filled with TSB and FTM and incubated at 24°C and 33°C for 14 days, respectively. Samples were considered contaminated (positive) when growth media appeared turbid at visual inspection as compared to sterile growth media (negative control), or when reported by the automatic system BacT/ALERT 3D. The presence of microorganisms in turbid samples was confirmed by subculture on agar plates or in fresh culture media.

### Detection of antibiotic residues in tissue homogenates and cryopreservation media

Six cryopreserved tissue samples and cryopreservation media for each bank-prepared antibiotic cocktail were evaluated for antibiotic content, as previously described [7]. The agar diffusion test was adapted for the detection of antibiotics in tissue homogenates and cryopreservation media samples using *Staphylococcus aureus* ATCC6538 (SA)-, *Pseudomonas aeruginosa* ATCC 9027 (PA)-, and *Candida albicans* ATCC10231 (CA)-seeded agar plates. Each tissue homogenate and cryopreservation medium was assessed at least in triplicate for each microbial strain. Cryopreservation media and homogenates of six tissues decontaminated with BASE.128 and cryopreserved according to CTBL method were used as a control. For both the direct inoculum and RESEP tube methods, antibiotic activity of tissue samples was evaluated immediately after tissue thawing and at the end of the 14-day sterility testing; antibiotic activity of cryopreservation media was assessed immediately after thawing and after inoculation in culture broths. Antibiotic-free cryopreservation medium and 10% BASE.128 solution plated on each microorganism seeded agar plate served as negative and positive control, respectively.

### Statistics

Tissue homogenates and cryopreservation media were grouped on the basis of antibiotic cocktail treatment (cocktail 1, cocktail 2, and BASE.128), and the mean inhibition zone diameters and standard errors were calculated for each microbial strain (SA, PA, CA).

## Results

### Presence of residual antibiotics in cryopreserved tissues and cryopreservation solutions

Immediately after thawing, all cryopreserved tissues homogenates, decontaminated with different antibiotic cocktails, induced similar inhibition zones in the range of 10.0–10.4 mm on SA-seeded plates. On the last day of sterility testing period, all tissues still induced 3.7–4.4 mm inhibition zones on SA plates.

**Figure 1:**
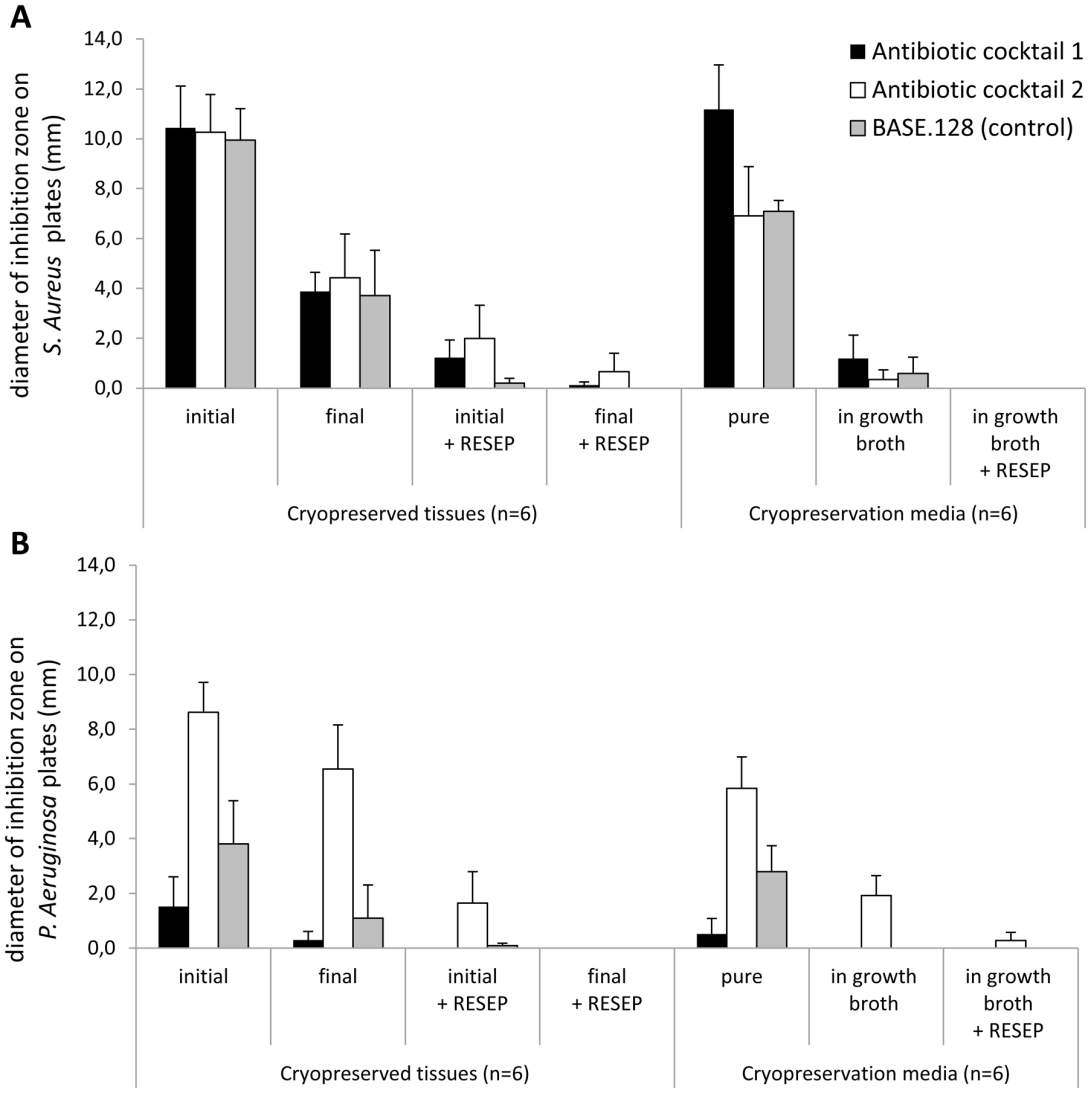
Presence of antibiotic residues in decontaminated and cryopreserved tissue homogenates and cryopreservation media. Agar diffusion assays performed using *Staphylococcus aureus-*seeded plates (A) and *Pseudomonas aeruginosa*-seeded plates (B), immediately after thawing (initial) and after 14 days of sterility testing (final) on tissues and media undergoing decontamination procedures with Cocktail 1 and Cocktail 2, prepared by tissue banks, and BASE.128 as a control. The plot legend on Figure 1A also applies to Figure 1B.

Antibiotic elimination from tissue samples with the RESEP tube reduced inhibition zones by 81%–98% and 94%–100% on initial and final day of the 14-day sterility testing period, respectively (Figure 1A). Similarly, all cryopreservation media inhibited the growth of SA on agar plates. Cocktail 1, cocktail 2, and BASE.128 residues induced 11.2-, 6.9-, and 7.1-mm inhibition zones on SA plates, respectively. All cryopreservation media inoculated in growth broths induced detectable SA inhibition activity that was totally abolished by the RESEP tube treatment (Figure 1A).

The width of inhibition zones on PA-seeded plates varied depending on the antibiotic cocktail used during tissue decontamination. Immediately after thawing, 1.5- and 8.6-mm zones were induced by cocktail 1- and cocktail 2-treated tissues, respectively. After the 14-day sterility testing period, cocktail 2-treated tissues still induced 6.6-mm inhibition zones, whereas cocktail 1- and BASE.128-treated tissues induced 0.3- and 1.1-mm inhibition zones on PA-seeded plates, respectively (Figure 1B).

The growth of PA on agar plates was also inhibited by all cryopreservation media. Cocktail 2 residues induced the largest inhibition zones (5.8 mm) on PA plates and 1.9-mm inhibition zones were still detected after inoculation of cryopreservation media in growth broths.

Inhibition zones of the RESEP tube-treated tissues and solutions were markedly reduced or absent on PA plates except for cocktail 2-treated tissues, which induced detectable inhibition zones despite the RESEP treatment (Figure 1B).

Inhibition zones were not detected on antimycotic-sensible CA plates for any investigated tissue homogenate or cryopreservation medium at any tested time point (Table S1).

### Microbiological analysis

Tissue bank microbiological analysis of tissue samples and transport solution showed that 50% of the tissues were initially contaminated. These mostly corresponded to the blood vessels donated after cardiac death, collected by CTBER and were detected in both tissue and liquid samples. In CTBL, which procured tissues only from brain death donors, the initial contamination rate was 30% and contaminants were recovered exclusively from transport liquid samples (Table 1). Approximately half of the contaminated tissues were contaminated with *S. epidermidis*. The remaining contaminants were represented by other bacterial strains, including *Enterobacter cloacae, Enterococcus faecalis, Escherichia coli, Streptococcus mitis, Streptococcus viridans*, *Corynebacterium spp. and Bacillus spp*. One tissue was contaminated with *C. albicans* (Table S2).

**Table 1:** Results of microbiological analysis.

		Positive samples							
		Before decontamination		After decontamination		After thawing			
		Direct inoculum		Direct inoculum		Direct inoculum		RESEP tube	
		tissue	liquid	tissue	liquid	tissue	liquid	tissue	liquid
CTBER (Tissue Bank 1)	n = 6	5/6	5/6*	1/6	1/6*	0/6	0/6	3/6	2/6
CTBL (Tissue Bank 2)	n = 10	0/10	3/10	0/10	0/10	0/10	0/10	5/10	3/10
Total	n = 16	5/16	8/16	1/16	1/16	0/16	0/16	8/16	5/16
	%	32%	50%	6%	6%	0%	0%	50%	32%

Positive (contaminated) samples determined by microbiological analysis performed by standard tissue bank methods and the RESEP tube method on tissue and liquid samples of human cardiovascular allografts processed by two cardiovascular tissue banks and tested before and after decontamination and after thawing.

* liquid samples were inoculated in BacT/ALERT blood culture vials.

After decontamination, 2 out of 6 samples tested by CTBER were still found positive for bacterial and fungal contaminants. One of these contaminations *(C. albicans)* was detected with the direct inoculum method on tissue sample and the other *(E. coli)* with the Bactalert method on liquid sample (Table 1). All samples tested by CTBL resulted negative after decontamination.

After thawing, the microbiological analysis of cryopreserved tissue samples and solutions performed with the direct inoculum method, i.e., in the presence of antibiotic residues, did not detect contamination in any of the tested tissue or liquid samples. Microbiological analysis of cryopreserved tissue samples and solutions performed after removal of antibiotic residues with the ResEP tube showed that 5 out of 10 (50%) CTBL samples and 3 out of 6 (50%) CTBER samples were positive. Altogether, RESEP method detected contaminations in 8 tissue samples and 5 related cryopreservation solutions (Table 1).

## Discussion

We used a fast and simple modified agar diffusion method that allowed us to detect rapidly the presence of antibiotics in tissue homogenates and cryopreservation media at different time points of sterility testingwithout the need for additional extraction methods. We showed that the significant levels of antibiotic residues can be detected in decontaminated cardiovascular tissues and processing liquids after thawing, despite rinsing and cryopreservation. In fact, antimicrobial activity against both gram-positive and gram-negative bacteria was detected by SA- and PA-seeded plates, which varied depending on decontamination cocktail and protocol. We speculate that inhibition zones induced by cocktail 2, particularly marked on PA-seeded plates, were due to different composition and higher concentration of antibiotics active against PA. The absence of inhibition zones on CA-seeded plates confirmed that the tested samples did not contain antimycotics, as expected for tissues treated with bank-prepared cocktails lacking such components. Similarly, no inhibition zones on CA-seeded plates were observed for control tissues treated with BASE.128, indicating no carry-over of amphotericin B deoxycholate in tissue and liquid samples.

Consistently with a previous paper [7], the antibiotics were not only present in tissue homogenates, but also transferred from tissues to cryopreservation liquids and sterility test growth broths, where they remained active for up to 14 days of sample incubation. This strongly argues against the assumption that antibiotics are efficiently degraded under the standard conditions of microbiological analysis. Moreover, although some antibiotics such as beta-lactam antibiotics (cefoxime and cefotaxime) are relatively labile, others, including polymyxin B, lincomycin, vancomycin, and gentamicin, are stable enough to induce bacteriostatic effects under these conditions, which is in accordance with our results.

Our study highlighted a possible issue of validity of the microbiological method used for detection of microbial contaminations in tissue banking due to the presence of antibiotic residues in the tested samples.

Tissue samples decontaminated with antibiotic cocktails are a specific type of microbiological sample that may present bacteriostatic and/or fungistatic properties [8], [15]. The intrinsic properties of the tissues, such as enzymatic activity, immunologic events, and presence of antimicrobial peptides [16], which are well described in the skin [17], cornea [18], and amniotic membrane [19], may be exacerbated by the presence of residual antibiotics following tissue decontamination with antibiotic cocktails [7], [8], [20]. The European and US Pharmacopoeias indicate eliminating any factor that may interfere with microbial growth during sterility testing from samples [9], [10].

In our study, the microbiological negativity of 14 out of 16 (88%) tissue and liquid samples was observed by standard tissue bank methods, after decontamination. The two bank-prepared cocktails used in the present study intentionally do not contain antimycotics and this may explain both the persistence of fungal contamination found in one tissue sample, despite the decontamination procedure, and its recovery by the direct inoculum method performed by the tissue banks. Fungal contaminations account for approximately 1% of total contaminations and correspond to the expected tissue discard rate of the two tissue banks.

All cryopreserved tissue and cryopreservation solution samples resulted negative with the direct inoculum method in the absence of antibiotics removal, whereas 50% of these samples resulted positive when tested with the RESEP tube method that comprises antibiotic residue removal. This clearly shows the false negative results obtained by the direct inoculum method.

Some automatic blood culture systems implement antibiotic neutralization features. However, they were specifically designed and validated for blood samples [13], [21], where the expected antibiotic concentrations are relatively low. Therefore, the reliability of such system is questionable, when used with tissue processing liquids containing higher concentrations of decontaminating agents [13], [15], [21].

Although the RESEP tube is specifically designed to remove antibiotics of BASE.128, it eliminated significant amounts of antibiotic residues from tissue and liquid samples decontaminated with all investigated cocktails.

In addition, the RESEP tube method allowed ascertaining that as much as 50% of the tissue samples were contaminated by bacteria and/or fungi, after decontamination and cryopreservation. Such contamination was not detected with the direct inoculum method. Our results confirm that latent contaminations may be masked by bacteriostatic and fungistatic concentrations of antimicrobial agents [22]. Validated rinsing procedures to remove antibiotic residues from tissue samples might enhance bacterial recovery [23]. The dilution of antibiotics under minimal inhibitory concentration is an acceptable approach [9]–[11]; however, it requires high volumes of growth media in case of high antibiotic concentrations.

The tissue banks determine allograft microbiological contamination indirectly, through the analysis of tissues samples and processing liquids. The heart valve banks normally analyze small pieces (1 cm^3^) of myocardium, aorta or pulmonary artery, and processing liquids with volumes ranging from 1 to 5 ml samples at different processing phases. These amounts usually correspond to less than 5% of the total tissue or processing liquid sample, and thus may not properly reflect the contamination profile of the allograft considering that at least 10% of the total volume for liquid samples should be tested according to European and US Pharmacopoeias [9], [10]. In fact, we observed that the results obtained in tissue and related liquid samples did not always match. This could be partially explained by low volume sampling and/or low bacterial concentration, at the limit of detection threshold in tissue, which is mainly expected in brain-dead donors and may lead to increased variability in results. We also speculated that there is a heterogeneous distribution of some microbial strains in specific tissue sites, which were not included among tested samples.

Furthermore, we observed that one contamination was detected after tissue decontamination, but not after tissue thawing using the direct inoculum method. This could be due to the sampling differences described above and/or decrease in the number of viable microorganisms under the detection threshold of the direct inoculum method during the last processing phase.

It may be argued that the microbiological positivity observed only in cryopreserved and the RESEP tube-treated samples could be a consequence of additional manipulation. However, none of the control samples undergoing identical manipulations were contaminated.

Microbiological analysis is closely related to tissue safety as results are used to determine the suitability of the tissue for transplantation [11]. Moreover, in case of falsely negative microbiological results, the inefficacy of the decontamination procedure is not immediately apparent and can potentially lead to the release of contaminated tissue, thus compromising the safety of the allograft recipient [15], [22].

The risks deriving from infected tissue transplants have been extensively documented [24], [25] and early postsurgical infections may have severe clinical implications [15], . In addition to antibiotic prophylaxis used to prevent the risk of surgical site infection [27], [28] and absence of immunosuppressant therapy, which is not routinely administered in tissue transplantation [3], [29], [30], thus maintaining immune defense against possible pathogens in allograft recipients, the elimination of false negative tissues could further improve the safety of transplantation procedures.

The policies concerning the management of microbiologically contaminated tissues differ considerably among Tissue Banks. Soo et al. discarded contaminated tissues before antibiotic treatment in order to not have false negatives, which probably results in higher than average discard rates [22].

Consistently with other reports [2], [14], [31], we observed higher contamination rate in donation after cardiac death donors as compared with brain-dead donors. This is likely due to agonal spread and postmortem translocation of microorganisms in cardiac death donors [31], [32], in which the time from death to cardiectomy is usually longer than that in brain-dead donors [14]. This highlights the need for an efficient decontamination procedure of tissues from these donors and process validation in terms of antibiotic cocktail composition, time and temperature incubation conditions, and related microbiological analysis.

Our data showed that approximately 50% of the cardiovascular tissues decontaminated by routine methods were microbiologically positive. Even if no adverse reactions have ever been reported after transplantation of cardiovascular allograft distributed by our banks, the results of the present study prompted us to review and optimize our decontamination protocols and microbiological methods.

## Conclusions

Antibiotic residues present in tissue samples and processing liquids after decontamination with antibiotic cocktails resulted in a high rate of falsely negative microbiological analysis. The RESEP tube method allowed a thorough elimination of residual antibiotics from tissue and processing liquid samples and a reliable microbiological assessment of tissue contamination. Although the results of the present study were observed in a relatively small group of tissues, they demonstrated the need for elimination of antibiotic residues from tested samples to improve the microbiological methods as required by current pharmacopeias.

## Supporting Information

Table S1 **Raw data of agar diffusion assays.**
(PDF)

Table S2 **Supplementary information on microbiological analysis.**
(PDF)
