# Supplementary material for: Residual Antibiotics in Decontaminated Human Cardiovascular Tissues Intended for Transplantation and Risk of Falsely Negative Microbiological Analyses
Source: PLoS One. 2014 Nov 14;9(11):e112679. doi: 10.1371/journal.pone.0112679 (PMC4232473; doi:10.1371/journal.pone.0112679)
Supplement: Table S1 — Raw data of agar diffusion assays. (PDF) [file pone.0112679.s001.pdf]

Agar Diffusion test inhibition zones

**ANTIBIOTIC COCKTAIL 1 treated tissues**			inhibition zones (mm) * induced by TISSUE HOMOGENATES						inhibition zones (mm) * induced by cryopreservation media				
	seeded microrganism	Plate n°	initial	final sample 1	final sample 2	initial + RESEP	final +RESEP sample 1	final + RESEP sample 2	pure	in growth broth sample 1	in growth broth sample 2	in growth broth + RESEP sample 1	in growth broth + RESEP sample 2
**TISSUE 1**	SA	p1	9,0	3,0	0,0	1,0	0,0	0,0	5,0	0,0	0,0	0,0	0,0
**aortic arch**	SA	p2	7,0	5,0	0,0	0,0	0,0	0,0	7,0	0,0	0,0	0,0	0,0
	SA	p3	10,0	nd	nd	0,0	nd	nd	7,0	0,0	0,0	0,0	0,0
	PA	p1	4,0	3,0	0,0	0,0	0,0	0,0	0,0	0,0	0,0	0,0	0,0
	PA	p2	3,0	4,0	0,0	0,0	0,0	0,0	0,0	0,0	0,0	0,0	0,0
	PA	p3	4,0	nd	nd	0,0	nd	nd	0,0	0,0	0,0	0,0	0,0
	CA	p1	0,0	0,0	0,0	0,0	0,0	0,0	0,0	0,0	0,0	0,0	0,0
	CA	p2	0,0	0,0	0,0	0,0	0,0	0,0	0,0	0,0	0,0	0,0	0,0
	CA	p3	0,0	nd	nd	0,0	nd	nd	0,0	0,0	0,0	0,0	0,0
**TISSUE 2**	SA	p1	11,0	3,8	3,7	5,0	0,0	0,0	9,0	2,0	1,0	0,0	0,0
**aortic arch**	SA	p2	8,0	4,0	4,0	4,0	0,0	0,0	11,0	3,0	2,0	0,0	0,0
	SA	p3	7,0	nd	nd	3,0	nd	nd	12,0	4,0	1,0	0,0	0,0
	PA	p1	0,0	0,0	0,0	0,0	0,0	0,0	0,0	0,0	0,0	0,0	0,0
	PA	p2	0,0	0,0	0,0	0,0	0,0	0,0	0,0	0,0	0,0	0,0	0,0
	PA	p3	0,0	nd	nd	0,0	nd	nd	0,0	0,0	0,0	0,0	0,0
	CA	p1	0,0	0,0	0,0	0,0	0,0	0,0	0,0	0,0	0,0	0,0	0,0
	CA	p2	0,0	0,0	0,0	0,0	0,0	0,0	0,0	0,0	0,0	0,0	0,0
	CA	p3	0,0	nd	nd	0,0	nd	nd	0,0	0,0	0,0	0,0	0,0
**TISSUE 3**	SA	p1	5,0	4,8	0,0	3,0	0,0	0,0	17,0	5,0	5,0	0,0	0,0
**femoral artery**	SA	p2	4,0	6,0	0,0	2,0	2,8	0,0	16,0	6,0	4,0	0,0	0,0
	SA	p3	5,0	nd	nd	1,0	nd	nd	15,0	5,0	5,0	0,0	0,0
	PA	p1	0,0	0,0	0,0	0,0	0,0	0,0	0,0	0,0	0,0	0,0	0,0
	PA	p2	0,0	0,0	0,0	0,0	0,0	0,0	0,0	0,0	0,0	0,0	0,0
	PA	p3	0,0	nd	nd	0,0	nd	nd	0,0	0,0	0,0	0,0	0,0
	CA	p1	0,0	0,0	0,0	0,0	0,0	0,0	0,0	0,0	0,0	0,0	0,0
	CA	p2	0,0	0,0	0,0	0,0	0,0	0,0	0,0	0,0	0,0	0,0	0,0
	CA	p3	0,0	nd	nd	0,0	nd	nd	0,0	0,0	0,0	0,0	0,0
**TISSUE 4**	SA	p1	13,0	3,6	3,5	0,0	0,0	0,0	10,4	0,0	0,0	0,0	0,0
**thoracic aorta**	SA	p2	13,2	4,2	4,2	0,0	0,0	0,0	10,1	0,0	0,0	0,0	0,0
	SA	p3	13,7	nd	nd	0,0	nd	nd	11,2	0,0	0,0	0,0	0,0
	PA	p1	0,0	0,0	0,0	0,0	0,0	0,0	0,0	0,0	0,0	0,0	0,0
	PA	p2	0,0	0,0	0,0	0,0	0,0	0,0	0,0	0,0	0,0	0,0	0,0
	PA	p3	0,0	nd	nd	0,0	nd	nd	0,0	0,0	0,0	0,0	0,0
	CA	p1	0,0	0,0	0,0	0,0	0,0	0,0	0,0	0,0	0,0	0,0	0,0
	CA	p2	0,0	0,0	0,0	0,0	0,0	0,0	0,0	0,0	0,0	0,0	0,0
	CA	p3	0,0	nd	nd	0,0	nd	nd	0,0	0,0	0,0	0,0	0,0
**TISSUE 5**	SA	p1	13,6	4,2	3,2	0,0	0,0	0,0	17,4	0,0	0,0	0,0	0,0
**femoral vein**	SA	p2	15,5	4,1	3,2	0,0	0,0	0,0	14,3	0,0	0,0	0,0	0,0
	SA	p3	14,5	nd	nd	0,0	nd	nd	15,4	0,0	0,0	0,0	0,0
	PA	p1	0,0	0,0	0,0	0,0	0,0	0,0	0,0	0,0	0,0	0,0	0,0
	PA	p2	0,0	0,0	0,0	0,0	0,0	0,0	0,0	0,0	0,0	0,0	0,0
	PA	p3	0,0	nd	nd	0,0	nd	nd	0,0	0,0	0,0	0,0	0,0
	CA	p1	0,0	0,0	0,0	0,0	0,0	0,0	0,0	0,0	0,0	0,0	0,0
	CA	p2	0,0	0,0	0,0	0,0	0,0	0,0	0,0	0,0	0,0	0,0	0,0
	CA	p3	0,0	nd	nd	0,0	nd	nd	0,0	0,0	0,0	0,0	0,0
**TISSUE 6**	SA	p1	12,6	8,3	6,0	0,4	0,0	0,0	7,6	0,0	0,0	0,0	0,0
**thoracic aorta**	SA	p2	13,0	6,4	7,6	2,0	0,0	0,0	8,2	0,0	0,0	0,0	0,0
	SA	p3	12,8	nd	nd	0,6	nd	nd	7,7	0,0	0,0	0,0	0,0
	PA	p1	5,3	0,0	0,0	0,0	0,0	0,0	2,9	0,0	0,0	0,0	0,0
	PA	p2	5,4	0,0	0,0	0,0	0,0	0,0	3,2	0,0	0,0	0,0	0,0
	PA	p3	5,6	nd	nd	0,0	nd	nd	3,2	0,0	0,0	0,0	0,0
	CA	p1	0,0	0,0	0,0	0,0	0,0	0,0	0,0	0,0	0,0	0,0	0,0
	CA	p2	0,0	0,0	0,0	0,0	0,0	0,0	0,0	0,0	0,0	0,0	0,0
	CA	p3	0,0	nd	nd	0,0	nd	nd	0,0	0,0	0,0	0,0	0,0

**ANTIBIOTIC COCKTAIL 2 treated tissues**			inhibition zones (mm) * induced by TISSUE HOMOGENATES						inhibition zones (mm) * induced by cryopreservation media				
	seeded microrganism	Plate n°	initial	final sample 1	final sample 2	initial + RESEP	final +RESEP sample 1	final + RESEP sample 2	pure	in growth broth sample 1	in growth broth sample 2	in growth broth + RESEP sample 1	in growth broth + RESEP sample 2
**TISSUE 1**	SA	p1	16,9	0,7	4,5	8,1	0,0	0,0	10,8	1,9	2,3	0,0	0,0
**aortic heart valve**	SA	p2	16,1	1,7	5,0	6,6	0,0	0,0	10,0	1,8	2,2	0,0	0,0
	SA	p3	17,1	nd	nd	6,5	nd	nd	10,5	2,1	2,3	0,0	0,0
	PA	p1	10,7	10,6	11,4	6,2	0,0	0,0	4,3	4,5	4,0	0,0	0,0
	PA	p2	11,0	10,9	11,4	5,2	0,0	0,0	4,1	3,8	3,4	0,0	0,0
	PA	p3	10,6	nd	nd	6,2	nd	nd	4,1	3,4	3,7	0,0	9,8
	CA	p1	0,0	0,0	0,0	0,0	0,0	0,0	0,0	0,0	0,0	0,0	0,0
	CA	p2	0,0	0,0	0,0	0,0	0,0	0,0	0,0	0,0	0,0	0,0	0,0
	CA	p3	0,0	nd	nd	0,0	nd	nd	0,0	0,0	0,0	0,0	0,0
**TISSUE 2**													
**thoracic aorta**	SA	p1	6,7	3,5	0,0	1,3	0,0	0,0	0,7	0,0	0,0	0,0	0,0
	SA	p2	9,0	4,0	0,0	0,4	0,0	0,0	0,5	0,0	0,0	0,0	0,0
	SA	p3	8,5	nd	nd	0,2	nd	nd	0,0	0,0	0,0	0,0	0,0
	PA	p1	6,0	4,2	0,0	0,0	0,0	0,0	2,4	0,0	0,0	0,0	0,0
	PA	p2	5,4	4,1	0,0	0,0	0,0	0,0	3,3	0,0	0,0	0,0	0,0
	PA	p3	5,4	nd	nd	0,0	nd	nd	3,3	0,0	0,0	0,0	0,0
	CA	p1	0,0	0,0	0,0	0,0	0,0	0,0	0,0	0,0	0,0	0,0	0,0

	CA	p2	0,0	0,0	0,0	0,0	0,0	0,0	0,0	0,0	0,0	0,0	0,0
	CA	p3	0,0	nd	nd	0,0	nd	nd	0,0	0,0	0,0	0,0	0,0
**TISSUE 3**													
**aortic heart valve**	SA	p1	5,1	11,7	11,6	0,0	4,4	3,4	4,9	0,0	0,0	0,0	0,0
	SA	p2	11,7	12,1	12,6	0,0	4,1	4,1	8,8	0,0	0,0	0,0	0,0
	SA	p3	nd	nd	nd	nd	nd	nd	nd	nd	nd	0,0	0,0
	PA	p1	11,1	5,4	5,9	4,5	0,0	0,0	8,3	2,7	3,2	0,0	0,0
	PA	p2	11,4	6,4	6,1	3,0	0,0	0,0	7,4	2,9	3,7	0,0	0,0
	PA	p3	12,7	nd	nd	3,9	nd	nd	12,0	5,4	5,8	0,0	0,0
	CA	p1	0,0	0,0	0,0	0,0	0,0	0,0	0,0	0,0	0,0	0,0	0,0
	CA	p2	0,0	0,0	0,0	0,0	0,0	0,0	0,0	0,0	0,0	0,0	0,0
	CA	p3	0,0	nd	nd	0,0	nd	nd	0,0	0,0	0,0	0,0	0,0
**TISSUE 4**													
**femoral vein**	SA	p1	8,5	2,9	0,0	0,0	0,0	0,0	13,7	0,0	0,0	0,0	0,0
	SA	p2	7,8	1,9	0,0	0,0	0,0	0,0	12,4	0,0	0,0	0,0	0,0
	SA	p3	8,4	nd	nd	0,0	nd	nd	12,7	0,0	0,0	0,0	0,0
	PA	p1	6,4	4,3	3,0	0,0	0,0	0,0	9,4	2,3	0,0	0,0	0,0
	PA	p2	6,5	4,3	3,0	0,0	0,0	0,0	8,7	2,3	0,0	0,0	0,0
	PA	p3	6,6	nd	nd	0,0	nd	nd	7,8	2,5	0,0	0,0	0,0
	CA	p1	0,0	0,0	0,0	0,0	0,0	0,0	0,0	0,0	0,0	0,0	0,0
	CA	p2	0,0	0,0	0,0	0,0	0,0	0,0	0,0	0,0	0,0	0,0	0,0
	CA	p3	0,0	nd	nd	0,0	nd	nd	0,0	0,0	0,0	0,0	0,0
**TISSUE 5**													
**saphenous vein**	SA	p1	11,6	2,6	5,2	4,1	0,0	0,0	6,4	0,0	0,0	0,0	0,0
	SA	p2	10,8	5,1	6,6	4,2	0,0	0,0	6,5	0,0	0,0	0,0	0,0
	SA	p3	11,5	nd	nd	4,4	nd	nd	6,0	0,0	0,0	0,0	0,0
	PA	p1	7,2	5,5	5,8	0,0	0,0	0,0	5,8	1,2	1,2	0,0	0,0
	PA	p2	8,2	8,5	4,9	0,0	0,0	0,0	6,1	0,7	0,7	0,0	0,0
	PA	p3	7,3	nd	nd	0,0	nd	nd	5,9	1,4	0,6	0,0	0,0
	CA	p1	0,0	0,0	0,0	0,0	0,0	0,0	0,0	0,0	0,0	0,0	0,0
	CA	p2	0,0	0,0	0,0	0,0	0,0	0,0	0,0	0,0	0,0	0,0	0,0
	CA	p3	0,0	nd	nd	0,0	nd	nd	0,0	0,0	0,0	0,0	0,0
**TISSUE 6**													
**aortic heart valve**	SA	p1	7,6	3,8	4,4	0,0	0,0	0,0	4,5	0,0	0,0	0,0	0,0
	SA	p2	9,3	2,6	3,6	0,0	0,0	0,0	4,7	0,0	0,0	0,0	0,0
	SA	p3	9,8	nd	nd	0,0	nd	nd	4,4	0,0	0,0	0,0	0,0
	PA	p1	8,7	10,9	11,1	0,4	0,0	0,0	3,7	1,2	2,1	0,0	0,0
	PA	p2	10,2	9,1	10,5	0,1	0,0	0,0	4,1	1,3	2,2	0,0	0,0
	PA	p3	9,7	nd	nd	0,1	nd	nd	4,4	1,4	nd	0,0	0,0
	CA	p1	0,0	0,0	0,0	0,0	0,0	0,0	0,0	0,0	0,0	0,0	0,0
	CA	p2	0,0	0,0	0,0	0,0	0,0	0,0	0,0	0,0	0,0	0,0	0,0
	CA	p3	0,0	nd	nd	0,0	nd	nd	0,0	0,0	0,0	0,0	0,0

**BASE.128 (CONTROL) treated tissues**			inhibition zones (mm)* induced by TISSUE HOMOGENATES						inhibition zones (mm)* induced by cryopreservation media				
Sample descritpion	seeded microrganism	Plate n°	initial	final sample 1	final sample 2	initial + RESEP	final +RESEP sample 1	final + RESEP sample 2	pure	in growth broth sample 1	in growth broth sample 2	in growth broth + RESEP sample 1	in growth broth + RESEP sample 2
**TISSUE 1**	SA	p1	11,5	4,7	5,4	0,0	0,0	0,0	7,5	0,0	0,0	0,0	0,0
**aortic heart valve**	SA	p2	11,7	5,3	4,4	0,1	0,0	0,0	7,7	0,0	0,0	0,0	0,0
	SA	p3	12,0	nd	nd	0,1	nd	nd	8,0	0,0	0,0	0,0	0,0
	PA	p1	7,5	0,0	0,0	0,0	0,0	0,0	5,1	0,0	0,0	0,0	0,0
	PA	p2	8,1	0,0	0,0	0,0	0,0	0,0	5,3	0,0	0,0	0,0	0,0
	PA	p3	7,7	nd	nd	0,0	nd	nd	6,0	0,0	0,0	0,0	0,0
	CA	p1	0,0	0,0	0,0	0,0	0,0	0,0	0,0	0,0	0,0	0,0	0,0
	CA	p2	0,0	0,0	0,0	0,0	0,0	0,0	0,0	0,0	0,0	0,0	0,0
	CA	p3	0,0	nd	nd	0,0	nd	nd	0,0	0,0	0,0	0,0	0,0
**TISSUE 2**													
**thoracic aorta**	SA	p1	10,8	4,2	4,4	0,0	0,0	0,0	6,0	0,0	0,0	0,0	0,0
	SA	p2	14,7	4,1	4,1	0,0	0,0	0,0	6,1	0,0	0,0	0,0	0,0
	SA	p3	10,5	nd	nd	0,0	nd	nd	6,2	0,0	0,0	0,0	0,0
	PA	p1	3,0	0,0	0,0	0,0	0,0	0,0	1,8	0,0	0,0	0,0	0,0
	PA	p2	3,2	0,0	0,0	0,0	0,0	0,0	1,6	0,0	0,0	0,0	0,0
	PA	p3	2,8	nd	nd	0,0	nd	nd	1,8	0,0	0,0	0,0	0,0
	CA	p1	0,0	0,0	0,0	0,0	0,0	0,0	0,0	0,0	0,0	0,0	0,0
	CA	p2	0,0	0,0	0,0	0,0	0,0	0,0	0,0	0,0	0,0	0,0	0,0
	CA	p3	0,0	nd	nd	0,0	nd	nd	0,0	0,0	0,0	0,0	0,0
**TISSUE 3**													
**aortic arch**	SA	p1	13,6	10,6	10,9	1,9	0,0	0,0	7,0	3,7	3,7	0,0	0,0
	SA	p2	13,7	11,0	10,8	0,5	0,0	0,0	6,7	3,2	3,2	0,0	0,0
	SA	p3	13,4	nd	nd	0,9	nd	nd	5,9	4,2	3,4	0,0	0,0
	PA	p1	3,9	0,0	0,0	0,0	0,0	0,0	1,3	0,0	0,0	0,0	0,0
	PA	p2	4,0	0,0	0,0	0,0	0,0	0,0	1,1	0,0	0,0	0,0	0,0
	PA	p3	4,2	nd	nd	0,0	nd	nd	1,1	0,0	0,0	0,0	0,0
	CA	p1	0,0	0,0	0,0	0,0	0,0	0,0	0,0	0,0	0,0	0,0	0,0
	CA	p2	0,0	0,0	0,0	0,0	0,0	0,0	0,0	0,0	0,0	0,0	0,0
	CA	p3	0,0	nd	nd	0,0	nd	nd	0,0	0,0	0,0	0,0	0,0
**TISSUE 4**													
**femoral vein**	SA	p1	7,2	0,0	0,0	0,0	0,0	0,0	10,4	0,0	0,0	0,0	0,0
	SA	p2	7,2	0,0	0,0	0,0	0,0	0,0	8,5	0,0	0,0	0,0	0,0
	SA	p3	6,7	nd	nd	0,0	nd	nd	7,2	0,0	0,0	0,0	0,0
	PA	p1	0,0	0,0	0,0	0,0	0,0	0,0	4,6	0,0	0,0	0,0	0,0
	PA	p2	0,0	0,0	0,0	0,0	0,0	0,0	4,2	0,0	0,0	0,0	0,0
	PA	p3	0,0	nd	nd	0,0	nd	nd	3,8	0,0	0,0	0,0	0,0
	CA	p1	0,0	0,0	0,0	0,0	0,0	0,0	0,0	0,0	0,0	0,0	0,0
	CA	p2	0,0	0,0	0,0	0,0	0,0	0,0	0,0	0,0	0,0	0,0	0,0
	CA	p3	0,0	nd	nd	0,0	nd	nd	0,0	0,0	0,0	0,0	0,0
**TISSUE 5**													
**thoracic aorta**	SA	p1	7,1	0,0	0,0	0,0	0,0	0,0	6,3	0,0	0,0	0,0	0,0
	SA	p2	8,0	0,0	0,0	0,0	0,0	0,0	6,5	0,0	0,0	0,0	0,0
	SA	p3	7,7	nd	nd	0,0	nd	nd	6,6	0,0	0,0	0,0	0,0
	PA	p1	0,0	0,0	0,0	0,0	0,0	0,0	0,0	0,0	0,0	0,0	0,0

	PA	p2	0,0	0,0	0,0	0,0	0,0	0,0	0,0	0,0	0,0	0,0	0,0
	PA	p3	0,0	nd	nd	0,0	nd	nd	0,0	0,0	0,0	0,0	0,0
	CA	p1	0,0	0,0	0,0	0,0	0,0	0,0	0,0	0,0	0,0	0,0	0,0
	CA	p2	0,0	0,0	0,0	0,0	0,0	0,0	0,0	0,0	0,0	0,0	0,0
	CA	p3	0,0	nd	nd	0,0	nd	nd	0,0	0,0	0,0	0,0	0,0
**TISSUE 6**													
**aortic heart valve**	SA	p1	7,0	4,4	4,8	0,0	0,0	0,0	6,7	0,0	0,0	0,0	0,0
	SA	p2	8,2	0,0	0,0	0,0	0,0	0,0	7,1	0,0	0,0	0,0	0,0
	SA	p3	8,1	nd	nd	0,0	nd	nd	7,2	0,0	0,0	0,0	0,0
	PA	p1	7,6	8,2	1,4	0,4	0,0	0,0	4,0	0,0	0,0	0,0	0,0
	PA	p2	8,3	9,3	7,5	0,4	0,0	0,0	4,3	0,0	0,0	0,0	0,0
	PA	p3	8,1	nd	nd	0,7	nd	nd	4,2	0,0	0,0	0,0	0,0
	CA	p1	0,0	0,0	0,0	0,0	0,0	0,0	0,0	0,0	0,0	0,0	0,0
	CA	p2	0,0	0,0	0,0	0,0	0,0	0,0	0,0	0,0	0,0	0,0	0,0
	CA	p3	0,0	nd	nd	0,0	nd	nd	0,0	0,0	0,0	0,0	0,0

SA *Staphylococcus aureus*
PA *Pseudomonas aeruginosa*
CA *Candida albicans*

* To determine inhibition zone diameter, the diameter of the wells (7 mm) was subtracted.
