# Supplementary material for: Residual Antibiotics in Decontaminated Human Cardiovascular Tissues Intended for Transplantation and Risk of Falsely Negative Microbiological Analyses
Source: PLoS One. 2014 Nov 14;9(11):e112679. doi: 10.1371/journal.pone.0112679 (PMC4232473; doi:10.1371/journal.pone.0112679)
Supplement: Table S2 — Supplementary information on microbiological analysis. (PDF) [file pone.0112679.s002.pdf]

**CTBER (Tissue Bank 1)**		**TISSUE BANK ANALYSIS**<br>*Tissues: direct inoculum in TSB and FTM, incubation at 35°C for 7 days*<br>*Liquids: direct inoculum in Bact/ALERT vials incubation at 37°C for 5 days*				**R&D ALCHIMIA ANALYSIS**<br>*direct inoculum in TSB and FTM, incubation at 24°C and 33°C for 14 days, respectively (EP).*			
		**Before decontamination**		**After decontamination**		**After Thawing (direct Inoculum)**		**After Thawing (RESEP tube)**	
**Sample description**		**Tissue**	**Liquid**	**Tissue**	**Liquid**	**Tissue**	**Liquid**	**Tissue**	**Liquid**
TISSUE 1	Aortic arch	P (*E. faecalis, C. albicans Corynebacterium spp.*)	P (*E. faecalis, C. albicans*)	P (*C. albicans*)	N	N	N	P	N
Negative Control 1	(TSB/FTM)					na	N	na	N
TISSUE 2	Aortic arch	P (*E. cloacae*)	P (*E. cloacae*)	N	N	N	N	P	P
Negative Control 2	(TSB/FTM)					na	N	na	N
TISSUE 3	Femoral artery	P (*E. coli, S. mitis*)	P (*E. coli*)	N	P	N	N	N	N
Negative Control 3	(TSB/FTM)					na	N	na	N
TISSUE 4	Thoracic aorta	N	N	N	N	N	N	N	N
Negative Control 4	(TSB/FTM)					na	N	na	N
TISSUE 5	Femoral vein	P (*S. epidermidis*)	P (S. epidermidis)	N	N	N	N	P	P
Negative Control 5	(TSB/FTM)					na	N	na	N
TISSUE 6	Aortic heart valve	P (S. epidermidis, Bacillus spp.)	P (*S. epidermidis, S. viridans*)	N	N	N	N	N	N
Negative Control 6	(TSB/FTM)					na	N	na	N

**CTBL (Tissue Bank 2)**		**TISSUE BANK ANALYSIS**<br>*direct inoculum in FTM , incubation at 37°C for 7 days*				**R&D ALCHIMIA ANALYSIS**<br>*direct inoculum in TSB and FTM, incubation at 24°C and 33°C for 14 days, respectively (EP).*			
		**Before decontamination**		**After decontamination**		**After Thawing (direct Inoculum)**		**After Thawing (RESEP tube)**	
**Sample description**		**Tissue**	**Liquid**	**Tissue**	**Liquid**	**Tissue**	**Liquid**	**Tissue**	**Liquid**
TISSUE 1	Aortic heart valve	N	N	N	N	N	N	P	P
Negative Control 1	(TSB/FTM)					na	N	na	N
TISSUE 2	Aortic heart valve	N	P (*S. epidermidis*)	N	N	N	N	P	N
Negative Control 2	(TSB/FTM)					na	N	na	N
TISSUE 3	Femoral vein	N	P (*S. epidermidis*)	N	N	N	N	N	N
Negative Control 3	(TSB/FTM)					na	N	na	N
TISSUE 4	Thoracic aorta	N	N	N	N	N	N	N	N
Negative Control 4	(TSB/FTM)					na	N	na	N
TISSUE 5	Thoracic aorta	N	P (*S. epidermidis*)	N	N	N	N	N	N
Negative Control 5	(TSB/FTM)					na	N	na	N
TISSUE 6	Saphenous vein	N	N	N	N	N	N	P	N
Negative Control 6	(TSB/FTM)					na	N	na	N
TISSUE 7	Aortic heart valve	N	N	N	N	N	N	P	P
Negative Control 7	(TSB/FTM)					na	N	na	N
TISSUE 8	Aortic heart valve	N	N	N	N	N	N	P	P
Negative Control 8	(TSB/FTM)					na	N	na	N
TISSUE 9	Thoracic aorta	N	N	N	N	N	N	N	N
Negative Control 9	(TSB/FTM)					na	N	na	N
TISSUE 10	Aortic heart valve	N	N	N	N	N	N	N	N
Negative Control 10	(TSB/FTM)					na	N	na	N

P	Positive (contaminated)
N	Negative (not contaminated)
TSB	Tryptone Soy Broth
FTM	Fluid Thioglycollate Medium
na	not applicable
EP	European Pharmacopoeia
